# The Prognostic Role of Cancer Stem Cells in Breast Tumors

**DOI:** 10.4021/jocmr1302w

**Published:** 2013-08-05

**Authors:** Maurizio Di Bonito, Monica Cantile, Gabriella Malzone, Giuseppina Liguori, Gerardo Botti

**Affiliations:** a Pathology Unit, Istituto Nazionale Tumori Fondazione “G. Pascale”, via Mariano Semmola 80131, Napoli, Italy

Recent acquisitions on human carcinogenesis suggest that small populations of tumor stem cells can influence and modify neoplastic cells behavior and aggressiveness, as well as therapeutic response. Breast cancer is a complex disease which encompasses a wide range of phenotypes with different histopathological, clinical and molecular features.

It is therefore extremely important to identify the unit of tumor cells which lead the progression of the disease in order to set the optimal therapy for each breast cancer subtype. Late studies advice that breast cancer ability to proliferate, progress and spread is based on a limited subpopulation of cells with properties similar to stem cells, which has led to coin the term “breast cancer stem cells” (BCSCs) [1]. The presence of BCSCs niches results in a poor sensitivity to current chemotherapy treatments. Therefore, the residual chemoresistant tumor stem cells would be able, after therapy, to fuel the development of a new tumor mass [2].

Several stemness markers have been described for the various histological subtypes of breast cancer, among them CD44, CD24, CD133, EpCAM, CD166, Lgr5, CD47, ALDH1 and the most recent ABCG2.

Trans-membrane proteins CD44 and CD24 are among the most studied BCSC markers.

High levels of CD44 associated to low levels of CD24 (CD44(+)/CD24(-/low)) would characterize stem populations in breast cancer [3].

Trans-differentiation of normal breast cells with CD44low/CD24+ phenotype, in BCSCs with CD44(+)/CD24(-/low) phenotype, is achieved after Ras oncogene activation along with TERT over-expression [4]. Either the absence or low expression of CD24 associated with CD44 high expression in BCSCs is related to an invasive phenotype, poor prognosis and low survival [5, 6].

Aldehyde dehydrogenase-1 (ALDH1) is a newly proposed marker for BCSCs identification.

ALDH1 high expression represents a marker for breast, lung, prostate, liver, neck and colon CSCs [7]. Specifically, ALDH1 expression in breast cancer accounts only for 20-25%. Of this percentage, an average of 5% of cells is positive to ALDH1. High positivity in tumor cells is associated with high histological grade, ERBB2 over-expression, absence of hormone receptors ER and PgR and worse prognosis [8, 9].

Recent and numerous studies show that positivity for CD133 allows to identify CSCs in breast cancer [10]. CD133 is expressed by several solid tumors, including invasive breast cancer triple negative, with very low levels of expression compared to other CSCs markers previously reported, like CD44 and ALDH1 [11]. In early-onset breast cancers, associated with mutations on BRCA1, CD133+ cells show CSCs properties [10]. The employment of this tumor stemness marker in breast cancers has become popular more recently and its expression is often described as associated with a worse prognosis [12, 13].

ABCG2, or Breast Cancer Resistance Protein (BCRP), recognizes and carries several conventional anti-tumor drugs, including small chemotherapy molecules. Numerous lines of evidence suggest that ABCG2 can be associated with the presence of tumor stem cells and correlates with chemoresistence and a poor prognosis in several human neoplasias [14]. ABCG2 was over-expressed in human hematopoietic stem cells (HSCs) and it proved to be a marker of stem cell populations in different organs, including pancreas, lung, testis, brain and prostate [15]. Its role in breast cancer is still fairly discussed, although recent lines of evidence have indicated ABCG2 as a potential stemness marker also in this neoplasia [16]. However, there are no important recommendations on its prognostic role in this malignancy.

The role of these markers in breast cancer progression is not clear yet and above all no markers have been defined yet as the most adequate for the characterization of the niches of tumor stem cells.

Likely, several combinations of these markers would allow a more appropriate characterization of CSCs in different breast tumor subtypes.

It should be also ascertained those most closely related to prognosis and to therapeutic treatment resistance. So, it could be possible to delineate a more correct stratification of patients at risk, speculating also to directly interfere with the activity of these molecules [17], as is the case in other malignancies [18, 19], in order to establish more personalized therapeutic strategies.
